# Phase 2 Clinical Trials of NA‐831 for the Treatment of Alzheimer’s Disease and Major Depressive Disorder, support the Neurogenesis Hypothesis

**DOI:** 10.1002/alz.095627

**Published:** 2025-01-09

**Authors:** Lloyd Tran, Zung Vu Tran, Fern Vu

**Affiliations:** ^1^ Biomed AI, Inc., San Jose, CA USA; ^2^ Biomed Industries, Inc., San Jose, CA USA

## Abstract

**Background:**

Adult Hippocampal Neurogenesis has been shown to be involved in Major Depressive Disorder (MDD) and Alzheimer’s disease (AD) pathology. NA‐831 is a small drug molecule, exhibiting neuroprotection, neurogenesis and memory enhancing properties.

**Method:**

For AD: A randomized clinical trial of NA‐831 was performed in 112 participants with mild and moderate Alzheimer’s disease (AD), half received the drugs and half received placebo. The patients with MCI received 10 mg of NA‐831 and patients with mild and moderate AD received 30 mg of NA‐831 or placebo orally per day.

Subjects with MCI met the NIA‐AA core criteria. CDR score of 0.5 and a Memory Box score of 0.5 or greater at Screening and Baseline. MMSE score ≥22. Subjects with mild & moderate AD to meet the NIA‐AA core criteria. MMSE: 17‐21.

For MDD: A randomized, clinical study was conducted to investigate the efficacy and safety of two fixed doses (20 and 40 mg/d) of NA‐831 *vs*. that of placebo after 6‐week treatment in 32 adult patients with major depressive disorder (MDD).

**Result:**

For AD: NA‐831 showed a significant improvement for patients with mild and moderate AD with the ADAS‐Cog‐13 score change of an average of 4.1 as compared to the placebo after 24 weeks of treatment (p = 0.001; ITT). CIBIC‐Plus showed 78% patients improved (p = 0.01; ITT). mNA‐831 was well‐tolerated at 30 mg/day. There were no serious adverse events observed.

For MDD: The difference between active treatment and placebo of ∼7 points on the MADRS translates into a clinically relevant difference in response rates of 32.5% units, compared to an average of 16% units for antidepressants approved by the USA FDA. Treatment with NA‐831 for 6‐week was well tolerated and efficacious in reducing depressive and anxious symptoms in patients with MDD.

**Conclusion:**

The Neurogenesis Hypothesis has been shown to be a viable approach for further research for Alzheimer’s disease and Major Depressive Disorder. Phase 3 trials of NA‐831 for the treatment of AD and MDD are being conducted. Results of the clinical trials will be provided as soon as they are available.

